# Interaction of Intestinal Bacteria with Human Rotavirus during Infection in Children

**DOI:** 10.3390/ijms22031010

**Published:** 2021-01-20

**Authors:** Roberto Gozalbo-Rovira, Antonio Rubio-del-Campo, Cristina Santiso-Bellón, Susana Vila-Vicent, Javier Buesa, Susana Delgado, Natalia Molinero, Abelardo Margolles, María Jesús Yebra, María Carmen Collado, Vicente Monedero, Jesús Rodríguez-Díaz

**Affiliations:** 1 Department of Microbiology, School of Medicine, University of Valencia, Avda. Blasco Ibáñez 17, 46010 Valencia, Spain; rovigoro@uv.es (R.G.-R.); anrucam@iata.csic.es (A.R.-d.-C.); cristina.santiso@uv.es (C.S.-B.); susana.vila@uv.es (S.V.-V.); javier.buesa@uv.es (J.B.); 2 Department of Biotechnology, Institute of Agrochemistry and Food Technology (IATA-CSIC), 46010 Valencia, Spain; yebra@iata.csic.es (M.J.Y.); mcolam@iata.csic.es (M.C.C.); btcmon@uv.es (V.M.); 3 Instituto de Investigación INCLIVA, Hospital Clínico Universitario de Valencia, 46010 Valencia, Spain; 4 Institute of Dairy Products of Asturias (IPLA-CSIC), 46010 Villaviciosa, Spain; sdelgado@ipla.csic.es (S.D.); natalia.molinero@csic.es (N.M.); amargolles@ipla.csic.es (A.M.); 5 Health Research Institute of Asturias (ISPA), 33300 Oviedo, Spain

**Keywords:** rotavirus, gut microbiota, *Ruminococcus*

## Abstract

The gut microbiota has emerged as a key factor in the pathogenesis of intestinal viruses, including enteroviruses, noroviruses and rotaviruses (RVs), where stimulatory and inhibitory effects on infectivity have been reported. With the aim of determining whether members of the microbiota interact with RVs during infection, a combination of anti-RV antibody labeling, fluorescence-activated cell sorting and 16S rRNA amplicon sequencing was used to characterize the interaction between specific bacteria and RV in stool samples of children suffering from diarrhea produced by G1P[8] RV. The genera *Ruminococcus* and *Oxalobacter* were identified as RV binders in stools, displaying enrichments between 4.8- and 5.4-fold compared to samples nonlabeled with anti-RV antibodies. In vitro binding of the G1P[8] Wa human RV strain to two *Ruminococcus gauvreauii* human isolates was confirmed by fluorescence microscopy. Analysis in *R. gauvreauii* with antibodies directed to several histo-blood group antigens (HBGAs) indicated that these bacteria express HBGA-like substances on their surfaces, which can be the target for RV binding. Furthermore, in vitro infection of the Wa strain in differentiated Caco-2 cells was significantly reduced by incubation with *R. gauvreauii.* These data, together with previous findings showing a negative correlation between *Ruminococcus* levels and antibody titers to RV in healthy individuals, suggest a pivotal interaction between this bacterial group and human RV. These results reveal likely mechanisms of how specific bacterial taxa of the intestinal microbiota could negatively affect RV infection and open new possibilities for antiviral strategies.

## 1. Introduction

Despite vaccination, group A rotavirus (RV) continues to be the leading etiologic agent of viral gastroenteritis in infants and young children worldwide [1] and is responsible for an estimated 130,000 deaths each year, mostly in developing countries [2]. A traditional dual-classification system of group A RV based on virion outer capsid proteins establishes more than 36 different G serotypes (depending on VP7 glycoprotein) and 51 P-types (depending on VP4 protease-sensitive protein) [3,4]. However, G1P[8], G2P[4], G3P[8] and G4P[8] RV are prevalent (over 90%) in most countries [5]. RV–host attachment is mediated by binding of the N-terminal portion of VP4 (VP8* fragment) to glycoconjugates at the cell surface. Diverse interactions between histo-blood group antigens (HBGA) and VP8* have been described, and, recently, the reported differences in the recognition of the fucosylated secretory H type-1 antigen versus its nonfucosylated precursor in the predominant P[8] group have explained the altered incidence of RV infections in secretor and nonsecretor individuals [6,7].

In the complex gut ecosystem where RVs develop, several interplays between the resident microbiota, the host glycobiology (e.g., mucosal HBGA) and enteric viruses have been described [6]. The classical view establishes that the gut microbiota protects against intestinal viral infections, but recent evidence also demonstrated a positive role for the microbiota in viral infection. Polioviruses and other intestinal viruses rely on the intestinal microbiota for infection by exploiting microbial-derived substances (e.g., lipopolysaccharide and peptidoglycan) to increase virion stability or to enhance attachment to host cells [8]. According to this, reduced viral infection is observed in gnotobiotic- or antibiotic-treated animal models [8,9,10,11], a situation that has also been observed for RV in a mouse model [11]. Some probiotic and commensal gut bacteria have the ability to in vitro bind RV and human noroviruses [12,13,14,15], and HBGA-like substances have been detected on the surface of enteric species such as *Enterobacter cloacae* [13], *Enterobacter faecium*, *Klebsiella* spp., *Citrobacter* spp. and *Hafnia alvei* [16]. The bacterium *E. cloacae* enhanced norovirus infectivity in gnotobiotic mice and in an in vitro model of infection in human B cells [10]. However, its application in gnotobiotic pigs reduced norovirus infectivity [17], for which some controversy still exists in this respect. The dual role of the microbiota in enteric virus infectivity (promoting or restricting) suggests that some microorganisms can be considered risk factors while others can lead to protection against infection. In agreement with this concept, diverse bacterial groups have been correlated to diminished or increased antibody titers (reflecting previous infections) against RV and norovirus [18]. An important breakthrough has been recently achieved after identifying that segmented filamentous bacteria (SFB), a group of microorganisms present in rodents and other vertebrates, and intimately associated with the intestinal epithelium, protect mice against RV [19].

Due to the increasing evidence of gut microbiota implication in RV infection, the aim of this work was to investigate which bacteria interact with RV during natural infant infections and their likely role in the process.

## 2. Results

### 2.1. Determination of Rotavirus Binding Bacteria by 16S rDNA Sequencing

**Figure 1:**
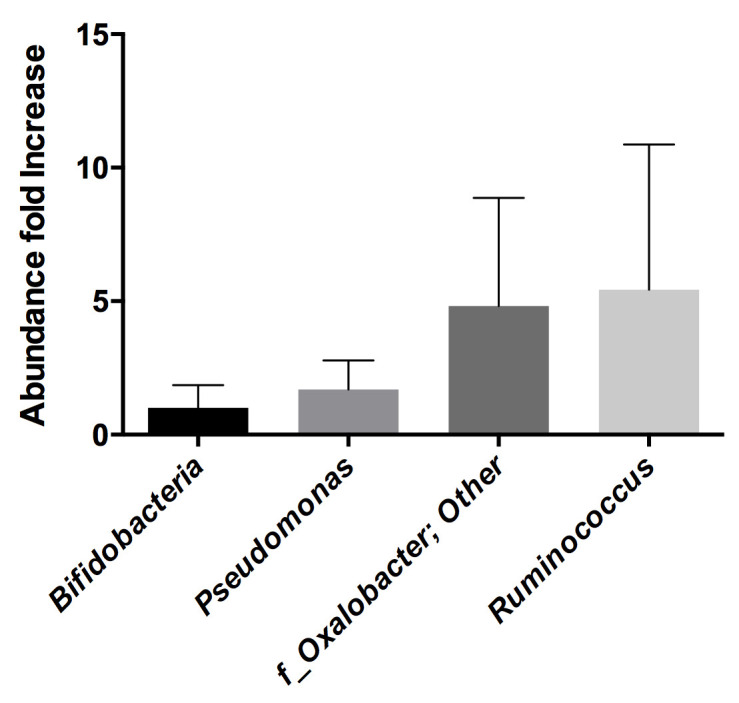
Increase in the relative abundance of several bacterial taxa at the genus level in the fluorescent channel of FITC-labeled bacteria (rotavirus-bound) compared to nonlabeled bacteria.

By using FACS coupled with a FITC-labeled anti-RV antibody, we identified bacteria interacting with RV in stool samples from five children suffering from RV diarrhea, clinically diagnosed as originating from the G1P[8] genotype. Total DNA was isolated from both sorted bacterial subpopulations (those RV-positive and RV-negative), and their microbial composition was determined by 16S rDNA sequencing. The information at the genus level was selected to analyze the differences in relative abundances of each bacterial group in the RV-binding and nonbinding bacteria. The *Ruminococcus* genus was identified as a RV binder, with percentages of abundance in the fluorescent versus the nonfluorescent bacterial groups showing a ratio of >5, followed by *Oxalobacter*, which presented a ratio of >4 (Figure 1). However, variability between samples was very high and the differences were mainly due to individual samples. In particular, samples from two different individuals accounted for most of the differences in *Ruminococcus* and *Oxalobacter* in the detected microbiotas.

### 2.2. Ruminococcus Gauvreauii Binds Rotavirus In Vitro

**Figure 2:**
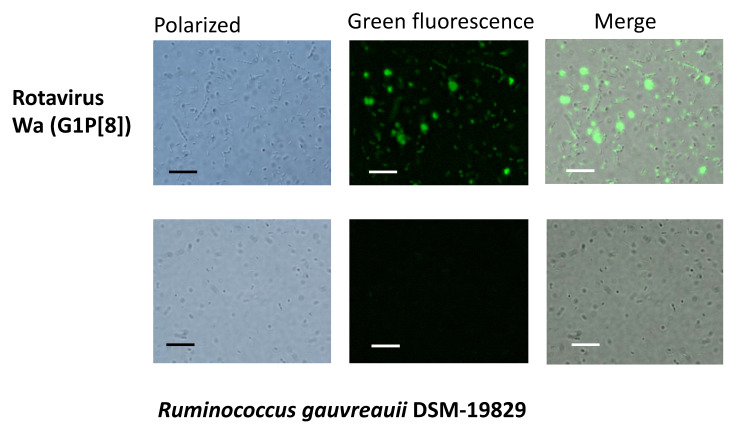
Microscopy images showing the interaction between bacteria from the genus *Ruminococcus* and rotavirus (RV) by fluorescence microscopy. Bacteria were incubated with the G1P[8] rotavirus strain Wa or without rotavirus (control), and images were collected in the polarized field and in green fluorescence. Merged images from both fields are also presented. The bars included as a reference possess a size of 10 μm.

The fact that the sequencing results revealed *Ruminococcus* as a bacterial genus with potential for RV binding attracted our attention for two different reasons: (i) this genus was previously linked to lower anti-RV antibody titers in adult humans [18], and (ii) the species *Ruminococcus gauvreauii* has recently been isolated from the human gall bladder [20]. We therefore analyzed the RV–*Ruminococcus* interaction using two strains of *R. gauvreauii*: the DSM-19829-type strain, isolated from human feces, and the IPLA-NM1 strain, a human bile isolate [20]. As a RV strain, we utilized Wa, a G1P[8] culture-adapted human RV with the same genotype as the pathogen present in the selected diarrheal samples. Both bacterial strains were able to bind Wa RV, as determined by fluorescence microscopy. The binding of RV to the *R. gauvreauii* DSM-19829 strain is shown in Figure 2. Interestingly, adding RV to the bacterial samples also favored a certain degree of bacterial clumping, indicating that bacteria agglutination can be induced with RV.

### 2.3. R. gauvreauii Expresses HBGA-Like Substances on Its Surface

**Figure 3:**
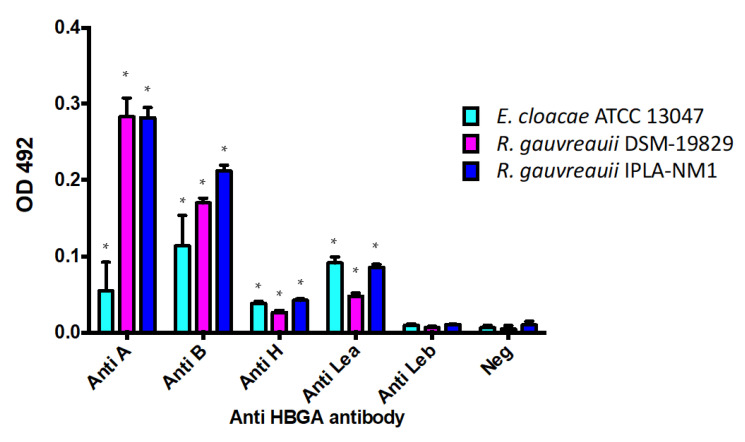
Histo-blood group antigens (HBGA)-like substances on the *Ruminococcus* surface detected by ELISA. *Ruminococcus gauvreauii* strains (DSM-19829 in purple and IPLA-NM1 in blue) and *Enterobacter cloacae* (positive control in light blue) were recognized by anti-A, anti-B, anti-H and anti-Lewis^a^ antibodies, indicating that these bacteria express HBGA-like substances on their surfaces. Neg.: negative control (no antibody). Statistical significance (*p* < 0.05) compared to the negative control is indicated by asterisks (*).

An ELISA assay was settled to determine whether RV-binding *R. gauvreauii* expresses HBGA-like substances on its surface, providing a mechanism for bacteria–RV physical interaction (Figure 3). Interestingly, the antibodies against the blood groups A and B and against the H-antigen and the Lewis^a^ antigen resulted positive for both *R. gauvreauii* strains tested (DSM-19829 and IPLA-NM1) as well as for the positive control *E. cloacae* ATCC 13047. No signal was detected with the anti-Lewis^b^ antibody. The performance of this antibody was confirmed in an ELISA with saliva from a Lewis^b^-positive secretor individual, as previously described [21], confirming that the bacteria did not express Lewis^b^-like substances on their surfaces. Interestingly, the ELISA results showed higher signals for some HBGA in *R. gauvreauii* strains compared to *E. cloacae*.

### 2.4. R. gauvreauii Interferes with Rotavirus Infection In Vitro

**Figure 4:**
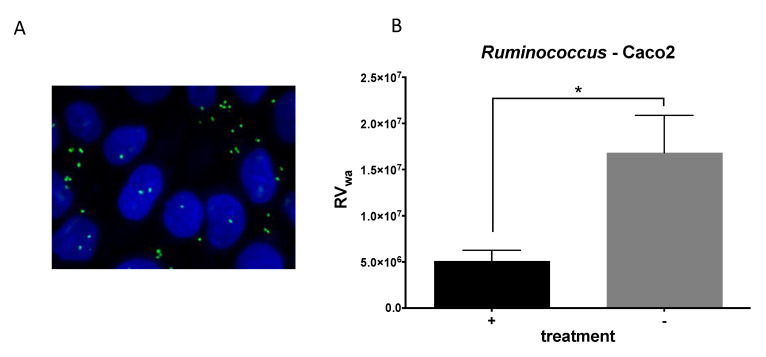
*Ruminococcus gauvreauii* and in vitro Wa infectivity. (**A**) *R. gauvreauii* IPLA-NM1 cells bound to the Caco-2 monolayer. Cells and bacteria were stained with DAPI, and several Z-stack images were taken and processed, as described in the Materials and Methods section (Section 4). Caco-2 nuclei are in blue, and *R. gauvreauii* cells are in green. (**B**) Inhibitory effect of preincubation of *R. gauvreauii* on Wa rotavirus infection measured as the reduction in viral genome equivalents in supernatants of Caco-2 cells after infection. Statistical significance (*p* < 0.05) compared to the negative control is indicated by asterisk (*).

Once the interaction between *R. gauvreauii* and RV was confirmed, the antiviral properties of this bacterium were tested. An infection assay was then carried out, in which the bacteria were preincubated with the cell monolayers before RV infection to simulate a natural infection, where the bacteria are pre-existing in the mucosal surface. Under these conditions, it was determined that *R. gauvreauii* IPLA-NM1 attaches to the Caco-2-cell-differentiated monolayer (Figure 4A), and a threefold decrease in Wa RV infectivity (measured as viral genome equivalents/mL of cell culture supernatants after infection) was found, demonstrating anti-RV activity for this bacterium (Figure 4B).

## 3. Discussion

Since the demonstration that the microbiota plays a key role in the infection of viruses targeting the intestine [8,22], much effort has been made to determine the role of gut bacteria in the relevant gastroenteritis-producing viruses, rotavirus and norovirus [6]. Unfortunately, despite the importance of this new concept, much of the evidence on the contribution of bacteria to RV infectivity has been achieved in cellular and animal models. Correlations between the abundance of several bacterial taxa and diminished antibody titers to norovirus and RV in adults have been reported [18], but data from infections in the relevant children group are still scarce and mainly derived from the results of seroconversion after vaccination with RV vaccine strains [23,24]. In this work, we contribute to filling this gap through the utilization of clinical samples from children with acute RV diarrhea. Using these samples, we identified *Ruminococcus* and *Oxalobacter* as two bacterial genera interacting with RV during diarrhea in two separate samples. One objection that could be argued against the idea that intestinal bacteria regulate the infectivity of RV is related to the different compartmentalization of RV replication and the intestinal microbiota. It is known that the gut microbiota mainly resides in the colon, while RV replication occurs in the small intestine [3], where bacterial numbers and diversity are much lower compared to the colon [25]. *Ruminococcus* and *Oxalobacter* are strictly anaerobic bacteria associated with the intestinal tract of mammals, and they can be isolated from human feces, indicative of a colonic habitat [26,27]. *Ruminococcus* species have been associated with the important function of degrading resistant starch from food [28,29], and they are important in the maturation of the intestinal microbiota during human development [30]. *Oxalobacter* is important for the metabolism of calcium oxalate in the intestine [31]. However, *Ruminococcus* has proved to reside also in the small intestine and gallbladder, as it can be isolated from bile [20]. The discovery of a *Ruminococcus*–RV interaction also coincided with the fact that intestinal *Ruminococcus* levels were shown to negatively correlate with IgA titers against RV [18]. We demonstrated P[8] RV surface binding in *R. gauvreauii*. This surface attachment can be the basis for the observed inhibitory effect of *R. gauvreauii* on the in vitro infectivity of the Wa RV strain. It was previously shown that enteric bacteria such as *E. cloacae* express A-, B- and H-like antigens on their surfaces and are able to bind human norovirus [13]. The same pattern of HBGA-like antigens was detected on the *R. gauvreauii* surface. The predominant P[8] RV interacted with the H type-1 antigen and A-type HBGA, while Lewis antigens were reported not to fit the binding site for these antigens [32]. However, a recent report pointed to a second carbohydrate-binding pocket for Lewis^b^ in this viral genotype [33]. H type-1 antigen and A-type HBGA binding could provide a mechanism by which RVs attach to the *R. gauvreauii* surface, suggesting that virus–bacteria adhesion could produce sequestration of viruses that impair viral infectivity. Previous experiments have shown a reduction in rotavirus infectivity in a similar setup as described here, such as, for instance, *Lactobacillus* and *Bifidobacterium* in Wa [34,35] or bovine RV infection in culture cells [36]. It has been recently shown that the chronic RV infection usually present in immunocompromised mice lacking B and T cells (Rag1-KO mice) can be prevented through ileal colonization by segmented filamentous bacteria (SFB; *Candidatus Arthromitus*) [19]. This protective effect does not involve immunoregulatory mechanisms triggered by SFB but probably depends on two nonmutually exclusive mechanisms: direct reduction of RV infectivity as a result of contact with SFB and enhanced enterocyte turnover. A recent study in a mouse model also evidenced a different role in RV infectivity for some bacterial groups. Infection by RV produces a shift in nutrient availability in the gut and induces goblet cells to release mucin, which promotes the growth of mucin-degrading *Bacteroides* and *Akkermansia* members in mice ileum. The consumption of mucin by these bacteria may, in turn, decrease the protection exerted by mucin against RV infection [37]. It is not known at this stage whether high *Ruminococcus* levels represent a protective factor against RV infection in children. In fact, samples where *Ruminococcus* were detected derived from children suffering from diarrhea. Nevertheless, *Ruminococcus* members are frequently found in the feces of children less than five years of age [38]. In a recent study, the microbiota in the feces from children suffering from RV diarrhea and those from healthy children were compared, and *Ruminococcus* was found significantly more abundant in healthy children, reinforcing the idea of a possible antiviral role of this bacterial genus [39] and confirming our previous RV–microbiota correlation studies [18].

More studies are needed to unravel the contribution of specific microbiota taxons to RV infection in humans. Some works have pointed to the relevance of differences in intestinal microbiota composition on the lack of efficacy (low vaccine take) of RV vaccines, a live viral vaccine, in low-income and middle-income settings [23,24], but this issue is far from being solved, and other possible contributing factors have been hypothesized [40]. Nevertheless, the bacteria–RV interacting pair here reported appears to be biologically relevant since the interaction was detected in natural infections and correlation of *Ruminococcus*–RV susceptibility was previously evidenced in human samples [18]. Our data constitute new insights into the bacteria-enteric viruses’ interplay during infection and could open new avenues for exploring innovative strategies to prevent and treat RV infections.

## 4. Materials and Methods

### 4.1. Rotavirus Detection and Genotyping

Stool samples from children younger than 5 years with diarrhea, who were admitted to the Hospital Clínico Universitario de Valencia in 2015, that resulted positive for RV via immunochromatography (Certest Biotec) were selected for genotyping. The genotyping of G and P genotypes of samples was performed following the European Network EuroRotaNet procedures (www.eurorotanet.com). Five stool samples with the G1P[1] genotype were selected and stored at −80 °C for further processing.

### 4.2. Preparation of Microbiota Samples from Stools and Cell Sorting

For detecting RV-binding bacteria, we followed a strategy similar to that recently described to quantify the in vitro binding of noroviral-virus-like particles to gut commensal bacteria [41]. Two-hundred milligrams of each stool sample was suspended in 1 mL of 0.9% saline solution (SS) by pipetting, followed by 10 s vortexing. The suspensions were centrifuged at 2000× *g* for 2 min to remove coarse materials, and the supernatants were further centrifuged at 12,000× *g* for 5 min to pellet the bacteria. The pelleted bacteria were washed twice in 1 mL of SS. The washed pellets were resuspended in 300 μL of SS and fixed by adding 1200 μL of 4% paraformaldehyde overnight at 4 °C. After fixation, the cells were washed 3 times with SS and finally resuspended in 1 mL of SS. One-hundred microliters were reserved to study the sample autofluorescence in the flow cytometer, and the other 900 μL were further processed for RV immunostaining. The cells were pelleted and suspended in 150 μL of SS containing 5% bovine serum albumin (BSA) (Sigma-Aldrich, St. Louis, MO, USA) for 1 h at 37 °C to prevent nonspecific antibody binding. After blocking, 1.5 µL of FITC-labeled goat anti-RV antibody (ab31435, Abcam) was added and incubated for 1 h at 37 °C. After RV labeling, 1.5 µL of a 1 mg/mL solution of propidium iodide (Thermo Fisher, Waltham, MA, USA) was added and incubated for 30 min at 37 °C for nucleic acid staining. Finally, the cells were pelleted, washed twice with SS and resuspended in 1 mL of SS.

**Figure 5:**
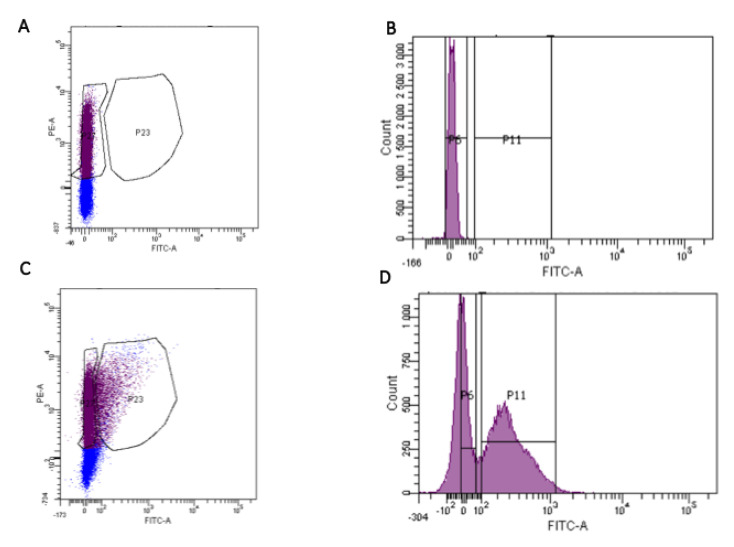
Fluorescence-activated cell sorting of rotavirus-bound fecal microbiota. Nonlabeled bacteria from the stools of patients were subject to FACS for determining autofluorescence and establishing the nonbinding population (**A**,**B**). The same bacteria were labeled with FITC-anti-rotavirus and subject to FACS (**C**,**D**). The P6 population contained the non-FITC fluorescent bacteria, and the P11 population contained the FITC fluorescent bacteria with the bound rotavirus. The bacteria (labeled and nonlabeled) were collected separately and analyzed by 16S rDNA sequencing.

Bacterial cells were sorted using a cytometer FACSAria™III Cell and BD FACSDiva v6.1.6 analysis software (BD Biosciences, San Jose, CA, USA) equipped with a two-laser system at 488 nm and 633 nm (Figure 5). Cell sorting was performed with a 70 μm nozzle. Microorganisms were determined using forward- and side-scatter parameters (FSC and SSC). A FITC detector (530/30 nm bandpass filter) was used to determine the levels of labeled bacteria (RV binders) and propidium iodide, and a PE detector (585/42 nm bandpass filter) was used to check for the presence of DNA. The parameter settings used comprised a SSC signal at 200 units as the threshold to define an event to be counted and voltages of FSC, SSC, FITC and PE of 16, 308, 350 and 455, respectively.

### 4.3. Sorted Fractions DNA Extraction and 16S rDNA Sequencing

Total DNA was isolated from the sorted bacteria by using the MasterPure Complete DNA & RNA Purification Kit (Epicentre) with a previous enzymatic step with 20 mg/mL of lysozyme (Roche) and 10 U/mL of mutanolysin (Sigma) at 37 °C for 1 h, followed by glass bead beating (0.17 mm diameter) for 1 min at 4 °C. The obtained DNA was amplified with the TruePrime WGA kit (Sygnis), following the manufacturer’s instructions. Total DNA concentration was measured and normalized using a Qubit^®^ 2.0 Fluorometer (Life Technology, Carlsbad, CA, USA). Subsequently, the V3-V4 region of the bacterial 16S rDNA gene was amplified by PCR using Illumina adapter overhang nucleotide sequences, following Illumina protocols. After 16S rDNA gene amplification, a multiplexing step was performed with the Nextera XT Index kit (Illumina, San Diego, CA, USA). Amplicons were checked with a Bioanalyzer DNA 1000 chip, and libraries were sequenced using a 2 × 300 bp paired-end run (MiSeq Reagent kit v3) on a MiSeq-Illumina platform (FISABIO Sequencing Service, Valencia, Spain). Controls during PCR amplification were also included and sequenced.

### 4.4. Bioinformatics and Statistical Analysis

Raw reads were searched for residual adaptors using the Trimmomatic program [42]. Artifacts, a quality check and quality trimming were performed with the prinseq-lite program [43]. R1 and R2 fastq reads were then joined using overlapping reads with the FLASH program [44]. The quality-filtered sequences were checked for chimera, and the nonchimeric sequences were processed using a QIIME pipeline (version 1.9.0) [45]. The sequences were clustered at 97% identity into operational taxonomic units (OTUs), and representative sequences were taxonomically classified based on the Greengenes 16S rRNA gene database (version 13.8). Sequences that could not be classified to the domain level or were classified as cyanobacteria and chloroplasts were removed from the dataset. Subsequently, to study the RV–microbiota interaction patterns, the threshold used for including the genera was 0.5% or greater in relative abundance in either the RV-positive or RV-negative fractions. The abundance of proportions of the given genera was log-transformed before calculating the ratio between the RV-positive or RV-negative fractions, resulting in the RV index. The index (calculated according to the formula log(RV++/RV−)) score reflects the degree of virus responsiveness and interaction to the specific microbial members, where the positive values represent the genera predominantly found to interact closely with RV, and the negative values represent the bacterial genera predominantly not interacting with RV. Calypso software (http://cgenome.net/calypso/) was used with total sum normalization (TSS) for the statistical analysis.

### 4.5. Bacterial Strains and Culture Conditions

*R. gauvreauii* DSM-19829 and *R. gauvreauii* IPLA-NM1 [20] strains were used in this study. *R. gauvreauii* DSM-19829 was purchased from the DSMZ Collection, and the IPLA-NM1 strain was isolated from a human bile sample [20]. They were grown in Gifu Anaerobic Medium (Nissui) supplemented with 0.25% (*w*/*v*) L-cysteine (Sigma-Aldrich) (named GAMc) incubated at 37 °C for 72 h in a Whitley MG500 anaerobic cabinet (Don Whitley Scientific) under a 10% H_2_, 10% CO_2_ and 80% N_2_ gas atmosphere. The two strains were routinely maintained by growing in 2% agar GAMc plates under anaerobic conditions.

### 4.6. Production of Rotavirus Infectious Viral Particles

The RV Wa strain, G1P[8], was cultured in MA104 cells and purified, as previously described [32]. Briefly, 10 confluent 150 cm^2^ flasks (approximately 1.5 × 10^7^ cells/flask) were infected with the Wa strain at a multiplicity of infection (MOI) of ≤0.1. One-hundred milliliters of medium with 1.5 × 10^8^ virus/mL were obtained, and the viral particles were concentrated by pelleting at 160,000× *g* for 1 h at 4 °C in an SW 41 rotor (Beckman). The viral pellet was resuspended in TNC buffer (20 mM Tris-HCl pH 8.0, 100 mM NaCl, 1 mM CaCl_2_) for obtaining triple-layered particles, and the particles were visualized by transmission electron microscopy after negative staining with phosphotungstic acid.

### 4.7. Ruminococcus–Rotavirus Binding Assay

Cells from *Ruminococcus gauvreauii* DSM-19829 and *R. gauvreauii* IPLA-NM1, an isolate from the human small intestine [20], were washed and diluted in PBS to an OD_595_ of 1. The cells were incubated with RV Wa at a concentration of 100 µg/mL of total viral antigen for 1 h at 37 °C with gentle agitation to allow the bacteria to interact with the virus in a final volume of 200 µL. After this period, the cells were centrifuged and washed two times with PBS to remove any unbound RV. The cells were resuspended in 200 µL of fixing solution (4% formaldehyde in PBS) and incubated for 15 min at 37 °C. After fixing, the bacterial suspensions were spread on glass microscope slides and air dried. The slides were blocked for 1 h at room temperature in PBS containing 1% BSA, and the viruses were detected by incubation with a mouse anti-RV IgG antibody raised in the laboratory, followed by an AlexaFluor488-labeled goat antimouse IgG antibody (1:400, Molecular Probes, Eugene, OR, USA). The preparations were mounted with 10 µL of the ProLong Gold Antifade Reagent (Life Technologies, Carlsbad, CA, USA) and visualized with an Eclipse 90i fluorescence microscope (Nikon, Tokyo, Japan). Controls without the virus and without the primary antibody (mouse anti-RV) were included.

### 4.8. HBGA ELISA on Bacterial Cells

An ELISA assay was settled up to determine whether the *Ruminococcus* strains expressed HBGA-like substances on their surface. ELISA plates (Costar) were coated with the two *R. gauvreauii* strains (DSM-19829 and IPLA-NM1). *Enterobacter cloacae* ATCC 13,047 (grown in nutrient broth at 37 °C overnight with shaking) was used as a positive control since it was previously determined that it expresses HBGA-like substances at its bacterial envelope [13]. The bacterial cells were washed three times in PBS and diluted to an OD_595_ of 1 in PBS. The wells of an ELISA plate were coated with 100 µL of the bacterial suspensions by incubation at 4 °C overnight. After coating, the wells were washed three times with 200 µL PBS containing 0.05% Tween 20 (PBS-T) and blocked with PBS-T containing 3% BSA for 1 h at 37 °C. After blocking, the plate was incubated for 1 h at 37 °C with monoclonal antibodies directed to HBGA (anti-A and anti-B, Diagast; anti-H, anti-Lea and anti-Leb, Covance, Princeton, NJ, USA) and diluted 1:100 in PBS with 1% BSA. After three washes, horseradish peroxidase goat antimouse IgG (Sigma) diluted 1:2000 in PBS-T plus 1% BSA was added and incubated for 1 h at 37 °C. After three washes with PBS-T, the reactions were developed with o-phenylenediamine dihydrochloride (OPD-Fast) (Sigma), stopped with 2M H_2_SO_4_ and recorded at 492 nm. The cut-off value was defined as a threefold increase in absorbance value compared to the negative control (cells incubated only with the secondary antibody). All the experiments were performed in triplicate.

### 4.9. Rotavirus Infection Blocking Assay with R. gauvreauii

Caco-2 cells were placed in 96-well cell culture plates (Costar) at 5 × 10^4^ cells/well in MEM medium containing 10% fetal bovine serum (FBS), 1% nonessential amino acids and 1× penicillin/streptomycin and allowed to differentiate for 11 days in a CO_2_ incubator at 37 °C, with culture medium changes every three days. After differentiation, cell monolayers were washed with serum-free medium. *R. gauvreauii* IPLA-NM1 cells were added at an OD_595_ of 0.01 to Caco-2 cells and incubated for 1 h at 37 °C. Control wells without added bacteria were included. Caco-2 monolayers were washed three times with serum-free medium containing 1 µg/mL of type IX trypsin (Sigma) and infected with activated rotavirus at 1.5 × 10^4^ genome equivalents/well (Wa RVs were previously activated by incubation in serum-free medium with type IX trypsin (Sigma) at 10 µg/mL for 30 min at 37 °C). RVs were kept in contact with the Caco-2 monolayers for 1 h at 37 °C. After this incubation period, the viral inoculum was removed, and the wells were washed twice with serum-free medium and incubated with serum-free medium containing 1 µg/mL of type IX trypsin (Sigma) for 16 h. After this period, the cell monolayers were disrupted with PBS containing 1% Triton X-100, and RNA was extracted using the Nucleospin-RNA virus Kit (Macherey-Nagel, Dylan, German), following the supplier instructions. The RV genome equivalents obtained after infection were calculated by RT-qPCR, as previously described [46]. Bacterial adhesion to the Caco-2 monolayers was also visualized by fluorescence. After incubation of the bacterial suspension with the Caco-2 monolayer, the cells were fixed and stained with DAPI. Nine 1 µm Z-stack images were taken at 100× using an Eclipse 90i microscope (Nikon Corporation, Tokyo, Japan). These image stacks were processed with Fiji (ImageJ 1.49q Software, National Institutes of Health, USA) and subdivided into two stacks, which contained basal visual information of the Caco-2 monolayer (mainly nuclei) and the bacteria located on the top, respectively. Both substacks were processed separately and finally merged into single images.

## 5. Conclusions

FACS, together with 16S rDNA NGS, are suitable technologies to unravel the members of the microbiota that physically interact with human rotaviruses in stool samples from infected children. Furthermore, bacteria from the genus *Ruminococcus* express HBGAs (putative rotavirus receptors) on their surfaces and possess antiviral activity in vitro.

## Data Availability

The dataset supporting the conclusions of this article is available in the NCBI’s Sequence Read Archive (SRA) repository, BioProject ID PRJNA676006 (http://www.ncbi.nlm.nih.gov/bioproject/676006).
